# Higher Incidence of Diabetes in Cancer Patients Compared to Cancer-Free Population Controls: A Systematic Review and Meta-Analysis

**DOI:** 10.3390/cancers14071808

**Published:** 2022-04-02

**Authors:** Keyi Yang, Zhunzhun Liu, Melissa S. Y. Thong, Daniela Doege, Volker Arndt

**Affiliations:** 1 Unit of Cancer Survivorship, Division of Clinical Epidemiology and Aging Research, German Cancer Research Center (DKFZ), 69120 Heidelberg, Germany; keyi.yang@dkfz-heidelberg.de (K.Y.); zhunzhun.liu@dkfz-heidelberg.de (Z.L.); m.thong@dkfz-heidelberg.de (M.S.Y.T.); d.doege@dkfz-heidelberg.de (D.D.); 2 Medical Faculty of Heidelberg, University of Heidelberg, 69120 Heidelberg, Germany

**Keywords:** cancer survivors, cancer patients, diabetes, cohort studies, systematic review, meta-analysis

## Abstract

**Simple Summary:**

Diabetes increases the risk of certain types of cancer. However, the literature regarding the incidence of diabetes after cancer diagnosis is inconsistent. We aimed to assess whether there was a higher incidence of diabetes among cancer patients by performing a systematic review and meta-analysis of results from cohort studies. To the best of our knowledge, this review conducted the largest and most up-to-date meta-analysis to compare the incidence of diabetes between cancer patients and the cancer-free population. Our results suggest that new-onset diabetes is positively associated with cancer.

**Abstract:**

Background: Diabetes increases the risk of certain types of cancer. However, the literature regarding the incidence of diabetes after cancer diagnosis is inconsistent. We aimed to assess whether there was a higher incidence of diabetes among cancer patients by performing a systematic review and meta-analysis of results from cohort studies. Methods: A systematic electronic literature search was carried out from cohort studies regarding the incidence of diabetes in cancer patients, using the databases PubMed (MEDLINE), Embase, Web of Science, and the Cochrane Library. Random-effects meta-analyses were conducted to pool the estimates. Results: A total of 34 articles involving 360,971 cancer patients and 1,819,451 cancer-free controls were included in the meta-analysis. An increased pooled relative risk (RR) of 1.42 (95% confidence interval (CI): 1.30–1.54, I^2^ = 95, τ^2^ = 0.0551, *p* < 0.01) for diabetes in cancer patients was found compared with the cancer-free population. The highest relative risk was observed in the first year after cancer diagnosis (RR = 2.06; 95% CI 1.63–2.60). Conclusions: New-onset diabetes is positively associated with cancer, but this association varies according to cancer type. More prospective studies with large sample sizes and longer follow-up times are advocated to further examine the association and the underlying mechanisms.

## 1. Introduction

Cancer today is considered a major public health problem globally. It is estimated that around 19.3 million new cancer cases were diagnosed in 2020 worldwide [1]. Due to improvements in cancer screening, diagnosis, and therapy as well as demographic aging [2,3,4], the number of cancer patients (including long-term cancer survivors who have survived for at least 5 years [5]) is increasing worldwide [6].

Previous studies have suggested that comorbid diabetes among cancer patients could be common [7]. Diabetes is found to be more prevalent in cancer patients than in the cancer-free population [8] which may be due to several reasons. For instance, cancer and comorbid diabetes could share common risk factors, such as older age, smoking, obesity, unhealthy diet, physical inactivity, and higher alcohol consumption [9]. Diabetes might also increase the risk for certain types of cancer such as breast cancer and colorectal cancer, and doubles the risk of liver, pancreas, and endometrial cancer [10]. In addition to the cancer disease, cancer treatments such as radiotherapy, glucocorticoids, targeted therapy, hematopoietic cell transplantation (HCT), and androgen deprivation therapy (ADT) may also result in an increased risk for diabetes [11,12,13,14,15,16,17,18,19].

In cancer patients, diabetes is associated with a poorer health-related quality of life (HRQOL) [20], higher healthcare utilization [21], and an increased risk of cancer progression and mortality [22,23,24], which highlights the clinical importance of knowing whether cancer patients are more likely to develop diabetes.

Currently, inconsistent results have been reported on the incidence of diabetes among cancer survivors compared with the cancer-free population. Several studies demonstrated a positive association between developing subsequent diabetes and cancer [25,26], while other studies did not [27]. A recent systematic review reported an overall positive association between cancer and incident diabetes by pooling 13 population-based cohort studies with time-to-event results, but that review did not include non-population-based studies and population-based studies without time-to-event data [28]. Therefore, in this study, we aimed to assess whether there was a higher incidence of diabetes among cancer patients and survivors by performing a systematic review and meta-analysis from the results of population-based and non-population-based cohort studies.

## 2. Materials and Methods

### 2.1. Electronic Searches

A comprehensive search was carried out for studies published from 1 January 2010 to 20 April 2021, using the databases PubMed (MEDLINE), Embase, Web of Science, and the Cochrane Library. The search was restricted to start from 2010, as there is a trend in increasing number of publications related to the aim of our study in recent years. Free-text words combined with the MeSH (for Pubmed and the Cochrane Library) and Emtree (for Embase) terms related to the review question were used: patients (cancer patients/survivors), controls (cancer-free population), outcomes (diabetes/comorbidity), and study type (cohort study). The detailed search strategy and specific terms are listed in Text S1 in Supplementary Material. In addition, a manual check on reference lists was also conducted to retrieve additional potentially relevant publications. Before data analyses, we conducted a second electronic search with an identical search strategy on 1 September 2021, to retrieve articles published since the first searching time point.

### 2.2. Inclusion Criteria

We included primary studies that met the following criteria: (1) conceptualized as prospective or retrospective cohort studies; (2) included a cancer-free control group; (3) included incident diabetes in the outcomes; (4) provided relative risk (RR), incidence rate ratio (IRR), hazard ratio (HR), or odds ratio (OR) with 95% confidence intervals (CI), or reported sufficient data to calculate these estimates; (5) were written in English; (6) were published as full-text in peer-reviewed journals. When more than one publication was reported on the same sample, the study with the most up-to-date or the most complete data was included.

### 2.3. Study Selection and Data Extraction

A consecutive process was conducted by KY to select primary articles according to the eligibility criteria: (1) duplicate check with Citavi 6^®^ (version 6.4.0.35, Swiss Academic Software, Wädenswil, Switzerland); (2) titles and abstracts screening; (3) full-text reading. After that, data were extracted by KY and ZL independently by using Microsoft Excel 2016 for Windows^®^. When there was a disagreement that could not be resolved after double checking between KY and ZL, MT was involved. Every step was confirmed by VA. Information was extracted from each of the included studies as follows: (1) basic information about the study (year of publication, first author, country of study, study design, study period, sample size, sample source, index date); (2) demographic characteristics of the case and control group (age at survey and diagnosis, and gender); (3) cancer site and method of diagnosis; (4) type of diabetes and method of diagnosis; (5) risk estimates with 95% CIs, confounders and adjustments.

### 2.4. Quality Assessment

Two reviewers (KY and ZL) independently used the nine-point Newcastle–Ottawa quality assessment scale (NOS) for cohort studies [29] to estimate the quality of each study included. The NOS is composed of eight items relating to three major domains: selection, comparability of cohorts, and the assessment of outcomes and follow-up. Each study could be awarded a maximum score of nine, and a score of 8 or 9 was defined as having a low risk of bias [30].

### 2.5. Statistical Analysis

The main outcome was the pooled RR in cancer patients compared with the cancer-free population. The HR, IRR, and OR were regarded as approximations of the estimates of the RR.

To obtain the pooled estimates, random-effects meta-analyses were conducted in this study. The inverse variance method was used for pooling, and the DerSimonian-Laird method was used to estimate the between-study variance (τ^2^). When no RR for the entire follow-up period was provided in a study, we first pooled the RR of each time interval using the method recommended by Tierney et al. [31]. Then the pooled estimate was used for the meta-analysis. When a study only contained several estimates from different subgroups but no overall RR, the estimate of each subgroup was directly added to the main analysis as the RR of an independent cohort if the case and controls had been matched within each subgroup. Otherwise, we calculated a combined RR for the subgroups using random-effects models and then added the combined RR into the main analysis.

The I² was calculated to estimate the proportion of the total variability that was due to between-study heterogeneity. We also calculated the 95% prediction interval which accounts for the uncertainty of the pooled estimate. Subgroup analyses stratified by study design (population-based or not), follow-up duration, gender, age at diagnosis, geographic regions, cancer type, diabetes type, specific factors controlled for (e.g., age, gender, prevalent comorbidities, body mass index (BMI)), and methodologic quality were carried out to explore the potential sources of heterogeneity. Sensitivity analyses were conducted by both omitting each one of the studies from the main analysis and including only studies reporting HRs, to examine whether results would significantly change. We also assessed potential publication bias using funnel plots combined with tests developed by Egger [32] and Begg [33]. A *p*-value < 0.1 in either Egger’s or Begg’s test would indicate the presence of publication bias.

The protocol of this study was predefined in accordance with the Preferred Reporting Items for Systematic Reviews and Meta-Analyses (PRISMA) checklist [34] and registered on PROSPERO (registration number CRD42021236041). All statistical analyses were performed using R software (version 4.1.1, R Core Team (2021), Vienna, Austria). All tests were two-tailed and *p*-values < 0.05 were considered statistically significant.

## 3. Results

### 3.1. Search and Identification of Studies

**Figure 1:**
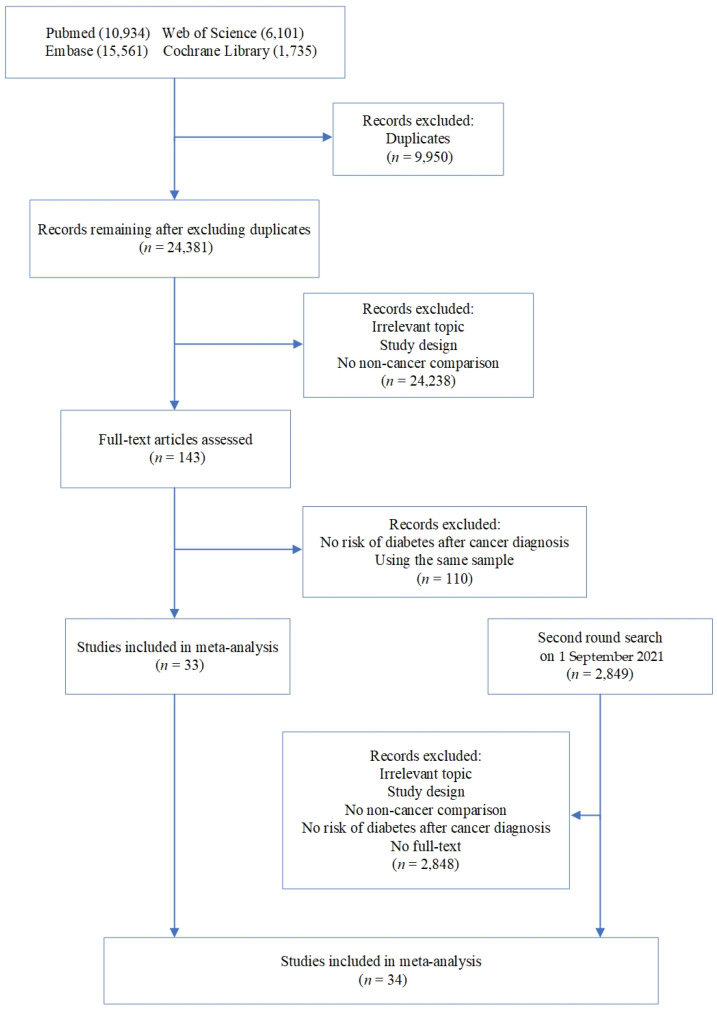
Flow chart for identification of studies.

A total of 34,331 articles were identified from initial electronic search. We excluded 9950 duplicates, and after title and abstract screening, the number of remaining references was reduced to 143. We then reviewed the full texts of the remaining literature to further rule out those that did not meet the inclusion criteria. The most frequent reasons for exclusion were irrelevant topics and the absence of cancer-free control groups. Details are shown in Figure 1. A second search was conducted shortly before the start of data analyses, and an additional 2849 articles were found. Finally, after a cross-reference check and screening, 34 articles were included in the meta-analysis.

### 3.2. Characteristics of Included Studies

The general characteristics of the 34 studies included are shown in Table 1 and Table S1 (in Supplementary Materials). The studies included were published between 2010 and 2021, with a total of 360,971 cancer patients (sample sizes ranged from 153 to 51,950) and 1,819,451 cancer-free controls (sample sizes ranged from 138 to 479,059) who reported no prevalent diabetes at the start of the study and were enrolled between 1991 and 2015. Over 60% of all the participants were female and most of the published papers were on breast cancer. These studies were performed in North America (18 of 34), Asia (7 of 34), Europe (6 of 34), and Australia (3 of 34). Almost all of the included studies were based on population-based cohorts (32 of 34). The information of the diagnosis of cancer and diabetes was obtained from cancer registries, public health organizations, or medical records. Six of the studies explicitly focused on type two diabetes mellitus (T2DM), and only one of the remaining studies differentiated between type one diabetes mellitus (T1DM) and T2DM in the analyses.

### 3.3. Quality Assessment of Included Studies

The risk of bias in each study included is shown in Table 2. In summary, most of the studies had a low risk of bias (27/34). Only one study indicated four areas of potential biases [45]. No clear statement of subjects lost to follow-up (13/34) was the most frequent source of potential bias, followed by insufficient length of follow-up (9/34).

### 3.4. Cancer Patients and the Association between Cancer and New-Onset Diabetes

**Figure 2:**
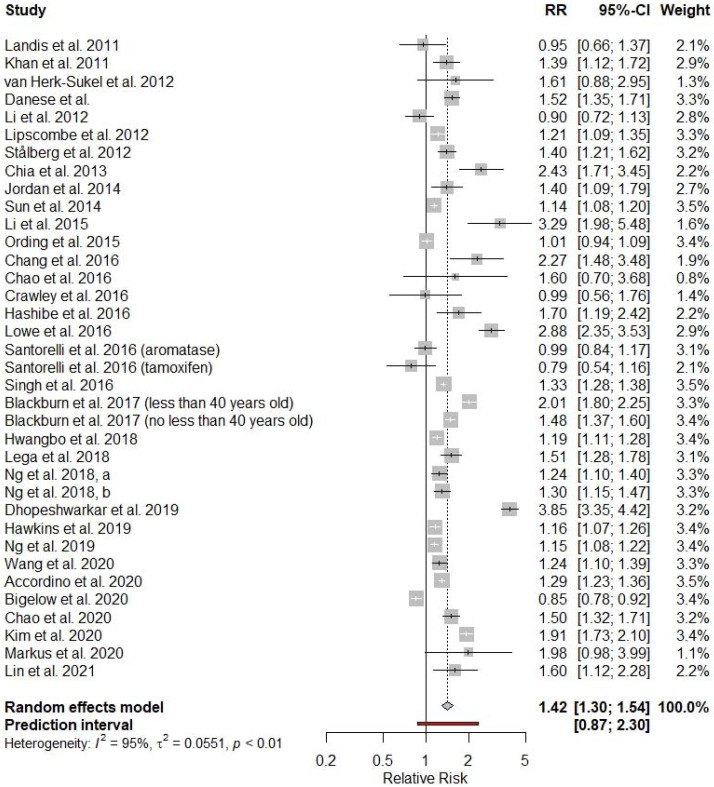
Overall relative risk of diabetes among cancer patients compared with cancer-free controls. The 95% prediction interval provides a predicted range for the true association between cancer and incident diabetes.

A total of 36 independent cancer cohorts from 34 studies were included in the main analysis. The pooled RR was 1.42 with a 95% confidence interval (CI) of 1.30 to 1.54. However, the between-study heterogeneity was high (τ^2^ = 0.0551, I^2^ = 95%, *p* < 0.01), and the 95% prediction interval was 0.87 to 2.30. In nine cohorts from eight individual studies, statistically nonsignificant results were reported, one study reported statistically significant inverse association between cancer and incident diabetes (HR = 0.85; 95% CI 0.78–0.92), and in all the other 26 cohorts from the other 25 studies, statistically significant positive associations were reported (Figure 2).

### 3.5. Subgroup Analyses and Sensitivity Analysis

We conducted subgroup meta-analyses stratified by various factors to further understand the association between cancer and incident diabetes in cancer patients and to investigate sources of heterogeneity. The results are shown in Table 3.

Consistent results were observed when stratified by study design (population-based or non-population-based), gender, age at diagnosis, study period, type of diabetes, risk of bias, and whether controlled for age, gender, prevalent comorbidity, or BMI. In subgroup analyses by cancer type, a statistically significant positive association between cancer and diabetes was found in most cancer types, except for tumors in the head and neck, central nervous system (CNS), prostate, gastric, uterus, esophagus, germ cell, and soft tissue sarcoma. Moreover, in the head and neck cancer subgroup, incident diabetes was inversely associated with cancer (RR = 0.86; 95% CI 0.80–0.93; I^2^ = 0), indicating that the head and neck cancer patients were less likely to have incident diabetes. Statistically nonsignificant associations were observed in some subgroups stratified by geographic regions (Europe, RR = 1.19; 95% CI 0.98–1.45), the time period post cancer diagnosis (>10 years, RR = 1.19; 95% CI 0.83–1.72), and treatment (endocrine therapy for breast cancer, RR = 1.11; 95% CI 0.99–1.26). I^2^s were not significantly reduced in most subgroup analyses. However, I^2^ = 0 was observed in some cancer type subgroups (head and neck, CNS, testicular). When stratified by therapy, the heterogeneity within the ADT subgroup was also small (I^2^ = 7).

**Figure 3:**
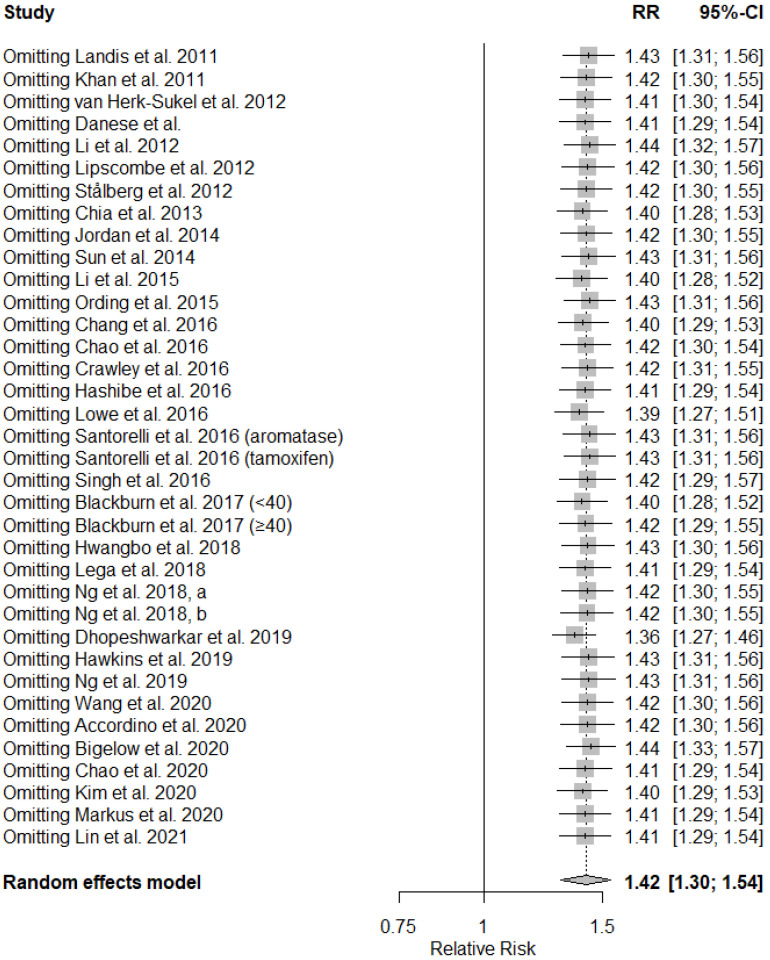
Sensitivity analysis.

In sensitivity analyses, the pooled RRs did not significantly alter when we omitted any of the studies from the main analysis, even those studies with a high risk of bias (Figure 3). When only the studies that reported HRs were included (*n* = 27), the results were similar to the original results (RR = 1.38; 95% CI 1.25–1.52). No statistically significant difference (*p* = 0.25) was observed when the pooled estimate of these 27 studies was compared with the pooled estimate of the non-time-to-event studies (*n* = 7; RR = 1.60; 95% CI 1.27–2.02).

### 3.6. Publication Bias

**Figure 4:**
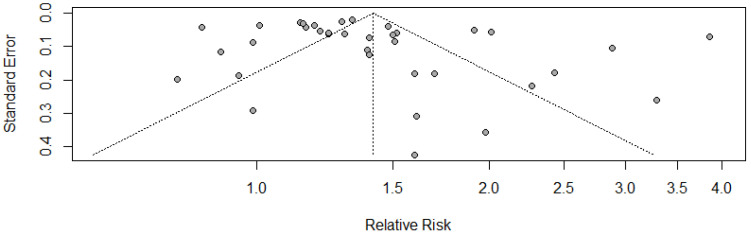
Funnel plot for publication bias.

The Egger’s test (*p* = 0.13) and the Begg’s test (*p* = 0.24) both indicated the absence of publication bias. However, the funnel plot seemed asymmetric (Figure 4), which might be attributed to the inherent heterogeneity between the included studies.

## 4. Discussion

To the best of our knowledge, this review conducted the largest and most up-to-date meta-analysis to compare the incidence of diabetes between cancer patients and the cancer-free population. In this review, we found that new-onset diabetes in general was positively associated with cancer during cancer survival, and there was a stronger association in the first year after cancer diagnosis. Xiao et al. [28] reported in their study an overall 1.39-fold increased risk of diabetes in cancer survivors when compared with the general population, with the pooled HR peaking in the first year post diagnosis (HR = 2.09; 95% CI 1.32–3.32), which is similar to our results. Consistent findings were also reported by other studies [8,11,66,67]. In a meta-analysis looking at the prevalence of metabolic syndrome in cancer patients compared with a cancer free population, a pooled OR of 1.08 (95% CI 0.57–2.03) for high glucose level was reported when stratified by individual components of metabolic syndrome. However, our meta-analysis included a greater number of, mostly large population-based cohort studies, and focused specifically on the incidence of diabetes. Therefore, our study is more likely to reveal the potential association between cancer and subsequently developed diabetes. Moreover, since diabetes could imply an increased risk for cardiovascular conditions such as coronary heart disease and stroke [68], our results are also consonant with findings that cancer patients are at a higher risk of cardiovascular disease (CVD) [69,70].

Although there is currently no clear explanation on the underlying mechanism for the association between cancer and diabetes, there are still some clues to be tracked. According to previous studies, most cancer treatment modalities could be positively associated with new-onset diabetes. When our body is not able to produce sufficient insulin or cannot use it effectively, diabetes occurs [71]. Interestingly, varying mechanisms for diabetes can be observed in different therapeutic methods according to cancer type. Surgery and radiotherapy involving the pancreas can result in pancreatic insufficiency, which is one of the important reasons for diabetes development [12]. Cranial irradiation and total body irradiation can influence the hypothalamic–pituitary axis, leading to changes in the body composition (e.g., overweight) and insulin resistance [13,14]. Although no evidence has been found that chemotherapeutic drugs could directly impinge on glucose metabolism, in classical chemotherapy regimens, they are often used in conjunction with glucocorticoids. Glucocorticoids can enhance the efficacy of chemotherapy, treat swelling, intracranial hypertension, pain, nausea, or be used as antitumor drugs in hematological malignancies [15]. Glucocorticoids affect several insulin-signaling pathways, leading to reduced insulin sensitivity, inducing insulin resistance, and increasing the risk of diabetes [15]. Targeted therapy drugs are also often used alone or in combination with chemotherapy. Some inhibitors block the pathways that are also involved in glucose regulation, such as the tyrosine kinase receptors insulin growth factor receptor 1 (IGF-1R) which could lead to insulin resistance [16]. Patients with hematologic malignancies are at high risk of exposure to glucocorticoids. They are also likely to receive HCT. HCT can contribute to the release of several proinflammatory cytokines such as interleukin-6 (IL-6) and tumor necrosis factor-α (TNF-α) [17,18], and previous studies have shown that the latter could contribute to insulin resistance by influencing the insulin signaling pathway [19]. ADT is widely used in prostate cancer [72]. A reduction in testosterone levels can be observed during the application of ADT drugs, however, decreased testosterone is directly associated with insulin resistance in men [73]. As for hormone therapy for breast cancer patients, we observed no significant association in a subgroup analysis involving only patients having received hormone therapy. This might be due to the favorable changes on the lipid profile by aromatase inhibitors (AIs) and tamoxifen [27,55].

In our current study, we found that the incidence of diabetes may be higher in the first year after cancer diagnosis. Closer contact between care providers and patients and a potential higher risk of detection bias in the initial years after cancer diagnosis might be possible explanations for this observation. Furthermore, some limitations in the underlying studies such as information bias regarding the correct assessment of the timing of the DM diagnosis and the potential survival bias during long-term follow-up could also explain these results. Moreover, since there is an age-related increase in diabetes risk in both cancer patients and controls [74], a reduced relative risk might be observed with the passing of time.

However, a statistically significant positive association was also observed during years one to ten. Several reasons could partly explain the fact that incident diabetes was still positively associated with cancer five or more years post diagnosis, when the effects of most treatments would most likely have subsided. First, in cancer patients, particularly those who suffer from cancer cachexia, insulin resistance often occurs as a result of the secretion and activation of several proinflammatory cytokines induced by cancer itself, such as TNF-α [75,76,77]. Some psychological consequences of cancer, such as depression, may also make the survivors more vulnerable to diabetes due to integrated mechanisms such as hypothalamic abnormality and an unhealthy lifestyle [78]. Moreover, the alteration of the glucose metabolism can start to appear more than ten years before the diagnosis of diabetes [79]. Furthermore, cancer and diabetes share a number of common risk factors, such as obesity, tobacco abuse, and alcohol consumption [9] and cancer survivors are more likely to be physically inactive compared with the general population [80]. A positive association was also observed ten or more years post diagnosis (Table 3). Although this result was not statistically significant (RR = 1.19, 95% CI 0.83–1.72), the potential positive association should not be ignored, and future studies with sufficient power and a sufficiently long follow-up post cancer diagnosis are recommended. Furthermore, extension of surveillance for incident diabetes to beyond ten years post diagnosis could be of clinical relevance.

In subgroup analyses, we found that the association between cancer and new-onset diabetes varied by cancer type. The highest relative risk of diabetes was found in pancreatic cancer, which was not surprising. This association in patients of hematologic malignancies was also strong, which was comparable with another study [81], possibly due to intensive and comprehensive therapy such as total body irradiation, chemotherapy with glucocorticoids, and HCT. However, we observed a statistically significant inverse association in the head and neck cancer patients. One possible explanation could be that cancer patients whose salivary glands and oral cavity were included in the treatment field, whether surgery or radiotherapy, could experience an unpleasant dietary intake thus would be less likely to become obese [82]. Furthermore, increased glucose-disposal rates were found in head and neck cancer patients indicating that the cancer cells played the role of a glucose drain [83]. However, the results were not always consistent. Future studies conducting research on head and neck cancer and diabetes should also assess covariates such as diet.

An increased risk of breast, colorectal, liver, and endometrial cancer has been detected among adults with diabetes [84]. In our study, new-onset diabetes was positively associated with these types of cancer. However, no statistical significance was found for uterine cancer based on two studies (RR = 1.56, 95% CI 0.97–2.50) (Table 3), which might be due to the small number of cases, the inclusion of other rare forms of cancer that arise in uterus (e.g., uterine sarcoma), and a possible bias from the exclusion of patients with prevalent diabetes at cancer diagnosis in the primary articles. For endometrial cancer alone, a statistically significant positive association was found in the primary article (HR = 1.91, 95% CI 1.73–2.10) [63].

Our study had some limitations. First, we focused on various types of cancer patients with no restriction on cancer treatment, age, gender, and follow-up duration, which could have contributed to the high heterogeneity of the included studies. In our study, reduced I^2^s were observed in some cancer type subgroups (head and neck, CNS, testicular) and the ADT subgroup (Table 3), which suggested that heterogeneity was generated by complex factors, and that cancer type and therapy could be two potential sources of heterogeneity. Since the study size in our meta-analysis was highly variable, and I^2^ is highly influenced by the size of the included studies [85], this could be another important reason for the high I^2^ reported. We also calculated the between-study variance (τ^2^ = 0.0551) and the 95% prediction interval (0.87–2.30) for the main analysis to analyze the heterogeneity. This indicated that there was substantial uncertainty about the significant association. It also provided a range for the association in a potential new original study on the association between new-onset diabetes and cancer overall. Furthermore, it offered a reference for clinicians to make more informed clinical decision making [86,87]. Second, apart from large population-based studies, we also included non-population-based studies with relatively smaller sample sizes. Nevertheless, the results in the main analysis, sensitivity analyses and subgroup analyses were mostly consistent, suggesting that our results of the underlying association are valid despite the high heterogeneity. Third, we could not perform more comprehensive subgroup analyses stratified by types of diabetes, treatment method, and other important factors such as BMI, physical activity, diet, smoking status, and alcohol consumption, due to the absence of individual patient data and due to a lack of studies that had differentiated relevant subgroups of cancer patients.

## 5. Conclusions

In summary, our meta-analyses of cohort studies revealed that new-onset diabetes was positively associated with cancer. This association was stronger during the first years after cancer diagnosis and varied according to cancer type. In this context, integration and coordination of healthcare should be applied for cancer patients. More prospective studies with large sample sizes and longer follow-up times (over ten years post diagnosis) are advocated to further examine the association and the underlying mechanisms.

## Figures and Tables

**Table 1:** Summary of the characteristics of studies included in meta-analysis (studies are sorted by year of publication and first author).

Study (First Author, Year of Publication, and Country)	Study Design	Study Period	Cancer Site	Participants		Association
				Cancer	Control	
Khan, 2011 (UK) [35]	PBC	2003–2006	BC, CRC, PC	26,276	104,486; 4:1, matched by age, gender and primary care practice	HR
Landis, 2011 (UK) [36]	PBC	2000–2007	SCCHN	1499	5996; 4:1, matched by age and gender	HR
Danese, 2012 (USA) [37]	PBC	1998–2002 ^a^	BC	51,950	51,950; 1:1, matched by time of diagnosis and geographic area	IRR ^b^
Li, 2012 (USA) [38]	PBC	1998–2008	PC	2616	2616; 1:1, matched by age, region of practice, length of follow-up, and observation period	OR
Lipscombe, 2012 (Canada) [39]	PBC	1996–2008	BC	24,976	124,880; 5:1, matched by age	HR
Stålberg, 2012 (Sweden) [40]	PBC	1993–2006	OC	11,139	55,687; up to 5:1, matched by birth year	HR
van Herk-Sukel, 2012 (The Netherlands) [41]	PBC	2000–2008	STS	533	5330; 10:1, matched by age and gender	HR
Chia, 2013 (USA) [42]	PBC	1998–2005	OC	5087	5087; 1:1, matched by the county of residence	IRR ^b^
Jordan, 2014 (USA) [43]	PBC	1990–2005	BC	1361	1361; 1:1, matched by age	HR
Sun, 2014 (Taiwan, China) [44]	PBC	2000–2011	BC	22,257	89,028; 4:1, matched by age and index year	HR
Li, 2015 (China) [45]	MCC	2008–2014	BC	1283	1361; not matched	RR ^b^
Ording, 2015 (Denmark) [46]	PBC	1999–2013	BC	32,403	162,015; 5:1, matched by year of birth and calendar date	RR ^b^
Chang, 2016 (China) [47]	PBC	2000–2012	AML	440	4400; 10:1, matched by age, gender, and index year	HR
Chao, 2016 (USA) [48]	PBC	1995–2010	Leukemia, Lymphoma, et al.	652	6520; 10:1, matched by age, gender, and calendar year of the index date	IRR
Crawley, 2016 (UK) [49]	PBC	2006–2013	PC	34,031	167,205; 5:1, matched by birth year and county of residence	HR
Hashibe, 2016 (USA) [50]	PBC	1991–2007 ^a.^	TeC	785	3323; 4-5:1, matched by birth year, birth region and the date of last residence in Utah	HR
Lowe, 2016 (USA) [51]	PBC	2000–2009	GC	12,612	12,612; 1:1, matched by year of enrollment in Medicare and county of primary residence	IRR ^b^
Santorell, 2016 (USA) [27]	PBC	2007–2009	BC	2678	10,712; 4:1, matched by age	HR
Singh, 2016 (Canada) [52]	PBC	2002–2012	CRC	39,707	198,535; 5:1, matched by age on the date of diagnosis and gender	HR
Blackburn, 2017 (USA) [53]	PBC	1997–2012 ^a^	ThC	3706	15,587; up to 5:1, matched by birth year, gender, and birth state	HR
Hwangbo, 2018 (South Korea) [25]	PBC	2002–2013	Pancreas, Kidney, et al.	15,130	479,059; not matched	HR
Lega, 2018 (Canada) [54]	PBC	1991–2015	Leukemia, Lymphoma, et al.	10,438	52,190; 5:1, matched by year of birth and gender	HR
Ng (BC), 2018 (Australia) [55]	PBC	2004–2014	BC	3799	37,990; 10:1, matched by age and gender	HR
Ng (PC), 2018 (Australia) [56]	PBC	2004–2014	PC	3176	31,760; 10:1, matched by age	HR
Dhopeshwarkar, 2019 (USA) [57]	PBC	2003-2013	AML	3911	3911; 1:1, matched by year of birth, gender, ethnicity, and geographic region	HR
Hawkins, 2019 (USA) [58]	PBC	1997–2013 ^a^	CRC	7114	25,979; up to 5:1, matched by birth year, gender, and birth state	HR
Ng, 2019 (Australia) [59]	PBC	2005–2013	BC	10,321	20,642; 2:1, matched by birth year	HR
Accordino, 2020 (USA) [60]	PBC	2005–2013	BC (including male BC)	13,529	13,529; 1:1, matched by date of birth and race	RR ^b^
Bigelow, 2020 (USA) [61]	PBC	2003–2013	SCCHN	2497	4994; 2:1, matched by age, race, gender, and SEER region	HR
Chao, 2020 (USA) [62]	PBC	2002–2014	Lymphoma, Melanoma, et al.	6778	87,737; 13:1, matched by age, gender, and calendar year	IRR
Kim, 2020 (USA) [63]	PBC	1997–2012 ^a^	EC	2314	8583; up to 5:1, matched by birth year, and birth state	HR
Markus, 2020 (Israel) [64]	SCC	2008–2015	MM (including SMM)	153	138; matched by age, gender, and length of follow-up	HR
Wang, 2020 (Taiwan, China) [65]	PBC	2000–2015	BC	4607	23,035; 5:1, matched by age	HR
Lin, 2021 (Taiwan, China) [26]	PBC	2000–2013	PC	1213	1213; 1:1, matched by index year, demographic variables, and comorbidities by propensity score matching	HR
Total	-	1991–2015	-	359,472	1,819,451	-

^a^ Participants were diagnosed with cancer during the listed time period. A time point for the end of the study was not given. ^b^ The estimate was not given directly, thus it was calculated with data provided in the primary article.

**Table 2:** Quality assessment of included studies using the Newcastle–Ottawa Scale (NOS) for cohort studies ^a^ (studies are sorted by year of publication and first author).

First Author	Selection				Comparability	Outcome			Total Score	Risk of Bias
	Representativeness of Cohort	Selection of Control Cohort	Ascertainment of Exposure	Outcome Not Present at Start	Comparability of Cohorts	Assessment of Outcome	Length of Follow-up	Adequacy of Follow-up ^b^	Maximum Score: 9	High (0–7) or Low (8–9)
Khan, 2011 [35]	*	*	*	*	**	*			7	High
Landis, 2011 [36]	*	*	*	*	**	*		*	8	Low
Danese, 2012 [37]	*	*	*	*	**	*		*	8	Low
Li, 2012 [38]	*	*	*	*		*	*	*	7	High
Lipscombe, 2012 [39]	*	*	*	*	**	*	*		8	Low
Stålberg, 2012 [40]	*	*	*	*	*	*			6	High
van Herk-Sukel, 2012 [41]	*	*	*	*	**	*		*	8	Low
Chia, 2013 [42]	*	*	*	*		*	*	*	7	High
Jordan, 2014 [43]	*	*	*	*	**	*	*	*	9	Low
Sun, 2014 [44]	*	*	*	*	**	*	*	*	9	Low
Li, 2015 [45]	*		*	*		*			4	High
Ording, 2015 [46]	*	*	*	*	**	*	*	*	9	Low
Chang, 2016 [47]	*	*	*	*	**	*	*	*	9	Low
Chao, 2016 [48]	*	*	*	*	**	*	*	*	9	Low
Crawley, 2016 [49]	*	*	*	*	**	*	*	*	9	Low
Hashibe, 2016 [50]	*	*	*	*	*	*	*	*	8	Low
Lowe, 2016 [51]	*	*	*	*		*		*	6	High
Santorell, 2016 [27]	*	*	*	*	**	*			7	High
Singh, 2016 [52]	*	*	*	*	**	*	*		8	Low
Blackburn, 2017 [53]	*	*	*	*	**	*	*	*	9	Low
Hwangbo, 2018 [25]	*	*	*	*	**	*	*		8	Low
Lega, 2018 [54]	*	*	*	*	*	*	*	*	8	Low
Ng (BC), 2018 [55]	*	*	*	*	*	*	*	*	8	Low
Ng (PC), 2018 [56]	*	*	*	*	*	*	*	*	8	Low
Dhopeshwarkar, 2019 [57]	*	*	*	*	**	*		*	8	Low
Hawkins, 2019 [58]	*	*	*	*	**	*	*		8	Low
Ng, 2019 [59]	*	*	*	*	**	*	*	*	9	Low
Accordino, 2020 [60]	*	*	*	*	**	*	*		8	Low
Bigelow, 2020 [61]	*	*	*	*	**	*	*		8	Low
Chao, 2020 [62]	*	*	*	*	**	*	*	*	9	Low
Kim, 2020 [63]	*	*	*	*	**	*	*	*	9	Low
Markus, 2020 [64]	*	*	*	*	**	*	*		8	Low
Wang, 2020 [65]	*	*	*	*	**	*	*		8	Low
Lin, 2021 [26]	*	*	*	*	**	*	*		8	Low

^a^ A study can be awarded a maximum of one point (*) for each item within the Selection and Outcome categories. A maximum of two points (**) can be given for Comparability. ^b^ We regarded a minimum of 5 years of follow-up time as sufficient length for follow-up.

**Table 3:** Subgroup analysis.

Subgroup	No. Studies	No. Estimates Pooled	RR (95% CI)	I^2^ (%)	Difference between Subgroups (*p*)
Overall	34	36	1.42 (1.30, 1.54)	95	-
Study design [sampling frame]	34	36			0.008
Population-based cohort	32	34	1.39 (1.28, 1.52)	95	
Non-population-based cohort	2	2	2.71 (1.67, 4.39)	24	
Gender	21	23			0.9169
Male	-	7	1.32 (1.09, 1.60)	63	
Female	-	16	1.31 (1.18, 1.45)	92	
Age at diagnosis	31	33			0.074
<50	-	8	1.64 (1.40, 1.93)	88	
≥50	-	25	1.36 (1.20, 1.55)	96	
Study region	34	36			0.0327
North America	18	20	1.49 (1.30, 1.70)	97	
Europe	6	6	1.19 (0.98, 1.45)	78	
Australia	3	3	1.21 (1.12, 1.31)	45	
Asia	7	7	1.38 (1.20, 1.58)	81	
Matched or adjusted for age and gender	34	36			0.1506
Yes	31	33	1.37 (1.25, 1.50)	95	
No	3	3	2.25 (1.15, 4.39)	97	
Matched or adjusted for status of prevalent comorbidity	34	36			0.2517
Yes	18	20	1.36 (1.22, 1.52)	96	
No	16	16	1.52 (1.30, 1.79)	93	
Matched or adjusted for BMI	34	36			0.5732
Yes	5	6	1.49 (1.23, 1.80)	96	
No	29	30	1.40 (1.27, 1.54)	95	
Follow-up length	34	36			0.7334
<5 years	5	6	1.51 (1.08, 2.12)	94	
5-10 years	14	14	1.35 (1.17, 1.56)	97	
>10 years	15	16	1.44 (1.27, 1.63)	93	
Time period post cancer diagnosis	15	29			0.0011
0–1 years	-	12	2.06 (1.63, 2.60)	95	
1–5 years	-	6	1.49 (1.31, 1.71)	90	
5–10 years	-	7	1.22 (1.08, 1.39)	76	
>10 years	-	4	1.19 (0.83, 1.72)	74	
Cancer entity	32	55			<0.0001
Pancreas	-	1	5.15 (3.32, 7.99)	-	
Hematologic	-	6	2.21 (1.52, 3.23)	87	
Kidney	-	1	2.06 (1.34, 3.16)	-	
Liver	-	1	1.94 (1.73, 2.10)	-	
Gallbladder	-	1	1.79 (1.08, 2.98)	-	
Testicular	-	2	1.74 (1.23, 2.46)	0	
Lung	-	1	1.72 (1.27, 2.32)	-	
Ovarian	-	3	1.63 (1.05, 2.53)	77	
Bladder	-	1	1.61 (1.08, 2.40)	-	
Thyroid	-	3	1.59 (1.26, 2.00)	92	
Breast	-	13	1.23 (1.14, 1.33)	86	
Colorectal	-	4	1.23 (1.10, 1.38)	79	
Gastric	-	2	1.97 (0.94, 4.13)	97	
Germ cell tumors	-	1	1.68 (0.98, 2.88)	-	
Soft tissue sarcoma	-	1	1.61 (0.88, 2.96)	-	
Uterus	-	2	1.56 (0.97, 2.50)	80	
Central nervous system		2	1.23 (0.80, 1.88)	0	
Prostate	-	5	1.10 (0.87, 1.41)	73	
Esophagus	-	1	0.83 (0.34, 1.99)	-	
Head and neck	-	4	0.86 (0.80, 0.93)	0	
Type of diabetes	34	37			0.1874
T1DM	-	1	3.93 (1.03, 14.94)		
T2DM	-	7	1.63 (1.16, 2.30)	98	
Not differentiated	-	29	1.37 (1.26, 1.48)	92	
Cancer therapy	11	15			0.0111
Chemotherapy	-	5	1.82 (1.22, 2.73)	92	
No chemotherapy	-	3	1.73 (1.24, 2.42)	96	
ADT	-	3	1.32 (1.15, 1.51)	7	
Endocrine therapy for breast cancer	-	4	1.11 (0.99, 1.26)	65	
Methodologic quality of study (NOS)	34	36			0.644
High (low risk of bias)	27	28	1.40 (1.28, 1.53)	96	
Low (high risk of bias)	7	8	1.51 (1.10, 2.08)	94	

## Supplementary Materials

The following supporting information can be downloaded at: https://www.mdpi.com/article/10.3390/cancers14071808/s1, Text S1: Searching strategies; Table S1: Summary of additional characteristics of studies included in meta-analysis.

## Abbreviations

ADT	Androgen deprivation therapy
AML	Acute myeloid leukemia
BC	Breast cancer
BMI	Body mass index
CI	Confidence interval
CNS	Central nervous system
CRC	Colorectal cancer
CVD	Cardiovascular disease
EC	Endometrial cancer
GC	Gastric cancer
HCT	Hematopoietic cell transplantation
HR	Hazard ratio
HRQOL	Health-related quality of life
IGF-1R	Insulin growth factor receptor 1
IL-6	Interleukin-6
IRR	Incidence rate ratio
MCC	Multi-center cohort
MM	Multiple myeloma
n.a.	not available
NOS	the Newcastle-Ottawa Scale for cohort studies
OC	Ovarian cancer
OR	Odds ratio
PBC	Population-based cohort
PC	Prostate cancer
PRISMA	Preferred Reporting Items for Systematic Reviews and Meta-Analyses
RR	Relative risk
SCC	Single center cohort
SCCHN	Squamous cell carcinoma of the head and neck
SMM	Smoldering multiple myeloma
STS	Soft tissue sarcoma
T1DM	Type 1 diabetes mellitus
T1DM	Type 2 diabetes mellitus
TeC	Testicular cancer
ThC	Primary thyroid cancer
TNF-α	Tumor necrosis factor-α
