# Supplementary material for: Higher Incidence of Diabetes in Cancer Patients Compared to Cancer-Free Population Controls: A Systematic Review and Meta-Analysis
Source: Cancers (Basel). 2022 Apr 2;14(7):1808. doi: 10.3390/cancers14071808 (PMC8997959; doi:10.3390/cancers14071808)
Supplement: Supplementary file 1 [file cancers-14-01808-s001.zip › cancers-1634413-supplementary.pdf]

cancers

Supplementary Materials

Higher Incidence of Diabetes in Cancer Patients Compared to Cancer-Free Population Controls: A Systematic Review and Meta-Analysis

Keyi Yang, Zhunzhun Liu, Melissa S. Y. Thong, Daniela Doege and Volker Arndt

Text S1. Searching strategies.

PUBMED (MEDLINE)

(((neoplasms[MeSH] OR neoplas*[tiab] OR malign*[tiab] OR cancer*[tiab] OR tumor*[tiab] OR tumour*[tiab] OR carcinoma*[tiab] OR "adenocarcinoma*"[tiab] OR "choriocarcinoma*"[tiab] OR "leukaemia*"[tiab] OR "leukemia*"[tiab] OR "lymphoma*"[Tiab] OR "sarcoma*"[Tiab] OR "teratoma*"[Tiab] OR "multiple myeloma*"[Tiab] OR "myeloma*"[Tiab] OR "melanoma*"[tiab]) AND (Surviv*[tiab] OR survival[MeSH] OR survivors[MeSH] OR ("after cancer"[Tiab] AND patient*[Tiab])) AND ((("long term"[Tiab] OR "long-term"[Tiab] OR "late"[Tiab]) AND "effect*"[Tiab]) OR Comorbidity[MeSH] OR "comorbid*"[Tiab] OR "co-morbid*"[Tiab] OR "polymorbid*"[Tiab] OR "poly-morbid*"[Tiab] OR "multimorbid*"[Tiab] OR "multi-morbid*"[Tiab] OR (("concomitant"[Tiab] OR "coexist*"[Tiab] OR "co-exist*"[Tiab] OR "chronic"[Tiab] OR "long-term"[Tiab] OR "long term"[Tiab]) AND ("illness*"[Tiab] OR "condition*"[Tiab] OR "disease*"[Tiab])) OR "DM"[Tiab] OR "IDDM"[Tiab] OR "NIDDM"[Tiab] OR "diabetes mellitus"[MeSH] OR diabet*[Tiab] OR "diabetes mellitus"[Tiab] OR "diabetes mellitus, type 1"[MeSH] OR "type 1 diabetes mellitus"[Tiab] OR "type 1 diabetes"[Tiab] OR "T1DM" OR "diabetes mellitus, type 2"[MeSH] OR "type 2 diabetes mellitus"[Tiab] OR "type 2 diabetes"[Tiab] OR "T2DM" OR "glucose intolerance"[MeSH] OR "glucose intoleran*"[Tiab] OR "insulin resistance"[MeSH] OR "insulin resistan*"[Tiab] OR "hyperglycemia"[MeSH] OR "hyperglycemia*"[Tiab] OR "hyperglycaemia*"[Tiab] OR "metabolic syndrome"[MeSH] OR "metabolic syndrome"[Tiab])) AND (risk[MeSH] OR risk*[Tiab] OR inciden*[Tiab] OR "incidence"[MeSH] OR "Odds Ratio"[MeSH] OR "ratio*"[Tiab]) AND ("General"[Tiab] OR "community" [Tiab] OR "cancer-free"[Tiab] OR "cancer free"[Tiab] OR "non-cancer"[Tiab] OR "non cancer"[Tiab] OR "no cancer"[Tiab] OR "health*"[Tiab] OR "compar*"[Tiab] OR "control*"[Tiab] OR "sibling*"[Tiab] OR "Control Groups"[MeSH]) AND ("cohort studies"[MeSH] OR "cohort"[All Fields] OR "longitudinal studies"[MeSH] OR "longitudinal"[All Fields] OR "follow-up studies"[MeSH] OR "follow-up"[All Fields] OR "follow up"[All Fields] OR "prospective studies"[MeSH] OR " prospective"[All Fields] OR "retrospective studies"[MeSH] OR "retrospective"[All Fields] OR "Observational Study"[Publication Type] OR "Observational"[All Fields])) NOT ("animals"[MeSH Terms] NOT "humans"[MeSH Terms]) AND (english[Filter])

EMBASE

#1 'neoplasm'/exp
#2 cancer* OR carcinoma* OR tumor* OR tumour* OR neoplasm* OR malign* OR oncolog* OR adenocarcinoma* OR choriocarcinoma* OR leukaemia* OR leukemia* OR lymphoma* OR sarcoma* OR teratoma* OR myeloma* OR 'melanoma' OR melanoma*:ab,ti
#3 #1 OR #2
#4 'cancer survivor'/exp OR 'cancer patient'/exp OR ((cancer*:ab,ti OR carcinoma*:ab,ti OR tumor*:ab,ti OR tumour*:ab,ti OR neoplasm*:ab,ti OR malign*:ab,ti OR oncolog*:ab,ti OR adenocarcinoma*:ab,ti OR choriocarcinoma*:ab,ti OR leukaemia*:ab,ti OR leukemia*:ab,ti OR lymphoma*:ab,ti OR sarcoma*:ab,ti OR teratoma*:ab,ti OR myeloma*:ab,ti OR 'melanoma':ab,ti OR melanoma*:ab,ti) AND surviv*:ab,ti) OR (((cancer* OR carcinoma* OR tumor* OR tumour* OR neoplasm* OR malign* OR oncolog* OR adenocarcinoma* OR choriocarcinoma* OR leukaemia* OR leukemia* OR lymphoma* OR sarcoma* OR teratoma* OR myeloma* OR 'melanoma' OR melanoma*) NEAR/5 (patient* OR 'man' OR 'men' OR 'woman' OR 'women' OR child*)):ab,ti)
#5 #3 AND #4
#6 'diabetes mellitus'/exp OR 'insulin dependent diabetes mellitus'/exp OR 'non insulin dependent diabetes mellitus'/exp OR 'glucose intolerance'/exp OR 'insulin resistance'/exp OR 'hyperglycemia'/exp OR 'metabolic syndrome x'/exp OR 'iddm'/exp OR 'niddm'/exp OR 't1dm'/exp OR 't2dm'/exp
#7 diabet* OR 'diabetes mellitus' OR dm OR iddm OR niddm OR 't1dm' OR 't2dm' OR 'type 1 diabetes mellitus' OR 'type 2 diabetes mellitus' OR 'type 1 diabetes' OR 'type 2 diabetes' OR hyperglycaemia* OR hyperglycemia* OR 'glucose intoleran*' OR 'insulin resistan*' OR 'metabolic syndrome':ab,ti
#8 'comorbidity'/exp OR ((('long term' OR 'long-term' OR 'late') NEXT/2 effect*) OR comorbid* OR 'co-morbid*' OR polymorbid* OR 'poly-morbid*' OR multimorbid* OR 'multi-morbid*' OR (('concomitant' OR coexist* OR 'co-exist*' OR 'chronic' OR 'long-term' OR 'long term') NEXT/2 (illness* OR condition* OR disease*)):ab,ti)
#9 #6 OR #7 OR #8
#10 'control group'/exp
#11 'general' OR ‘community’ OR 'cancer-free' OR 'cancer free' OR 'non-cancer' OR (('free' OR 'no' OR 'non' OR 'not' OR 'without') NEAR/5 (cancer* OR carcinoma* OR tumor* OR tumour* OR neoplasm* OR malign* OR oncolog* OR adenocarcinoma* OR choriocarcinoma* OR leukaemia* OR leukemia* OR lymphoma* OR sarcoma* OR teratoma* OR myeloma* OR 'melanoma' OR melanoma*)) OR 'health*' OR 'compar*' OR 'control*' OR 'sibling*':ab,ti
#12 #10 OR #11
#13 'incidence'/exp OR 'risk'/exp OR 'ratio'/exp OR 'hazard ratio'/exp OR 'odds ratio'/exp OR 'risk ratio'/exp
#14 inciden* OR risk* OR ratio*:ab,ti
#15 #13 OR #14
#16 'cohort analysis'/exp OR 'longitudinal study'/exp OR 'follow up'/exp OR 'prospective study'/exp OR 'retrospective study'/exp OR 'observational study'/exp
#17 ('cohort' OR 'longitudinal' OR 'follow-up' OR 'follow up' OR 'prospective' OR 'retrospective' OR 'observational') NEAR/5 (‘study’ OR ‘studies’ OR design* OR research* OR trial*)
#18 #16 OR #17
#19 #5 AND #9 AND #12 AND #15 AND #18
#20 [humans]/lim AND [english]/lim
#21 #19 AND #20

Cochrane Library

#1 MeSH descriptor: [Neoplasms] explode all trees
#2 MeSH descriptor: [Carcinoma] explode all trees
#3 MeSH descriptor: [Adenocarcinoma] explode all trees
#4 MeSH descriptor: [Choriocarcinoma] explode all trees
#5 MeSH descriptor: [Leukemia] explode all trees
#6 MeSH descriptor: [Lymphoma] explode all trees
#7 MeSH descriptor: [Sarcoma] explode all trees
#8 MeSH descriptor: [Teratoma] explode all trees
#9 MeSH descriptor: [Multiple Myeloma] explode all trees
#10 MeSH descriptor: [Melanoma] explode all trees
#11 (cancer* OR carcinoma* OR tumor* OR tumour* OR neoplasm* OR malign* OR oncolog* OR adenocarcinoma* OR choriocarcinoma* OR leukaemia* OR leukemia* OR lymphoma* OR sarcoma* OR teratoma* OR myeloma* OR 'melanoma' OR melanoma*):ab,ti,kw
#12 #1 OR #2 OR #3 OR #4 OR #5 OR #6 OR #7 OR #8 OR #9 OR #10 OR #11
#13 MeSH descriptor: [Cancer Survivors] explode all trees
#14 (((cancer* OR carcinoma* OR tumor* OR tumour* OR neoplasm* OR malign* OR oncolog* OR adenocarcinoma* OR choriocarcinoma* OR leukaemia* OR leukemia* OR lymphoma* OR sarcoma* OR teratoma* OR myeloma* OR 'melanoma' OR melanoma*) AND surviv*) OR ((cancer* OR carcinoma* OR tumor* OR tumour* OR neoplasm* OR malign* OR oncolog* OR adenocarcinoma* OR choriocarcinoma* OR leukaemia* OR leukemia* OR lymphoma* OR sarcoma* OR teratoma* OR myeloma* OR 'melanoma' OR melanoma*) NEAR/5 (patient* OR 'man' OR 'men' OR 'woman' OR 'women' OR child*))):ab,ti,kw
#15 #13 OR #14
#16 #12 AND #15
#17 MeSH descriptor: [Diabetes Mellitus] explode all trees
#18 MeSH descriptor: [Diabetes Mellitus, Type 1] explode all trees
#19 MeSH descriptor: [Diabetes Mellitus, Type 2] explode all trees
#20 MeSH descriptor: [Glucose Intolerance] explode all trees
#21 MeSH descriptor: [Insulin Resistance] explode all trees
#22 MeSH descriptor: [Hyperglycemia] explode all trees
#23 MeSH descriptor: [Metabolic Syndrome] explode all trees
#24 (diabet* OR 'diabetes mellitus' OR dm OR iddm OR niddm OR 't1dm' OR 't2dm' OR 'type 1 diabetes mellitus' OR 'type 2 diabetes mellitus' OR 'type 1 diabetes' OR 'type 2 diabetes' OR hyperglycaemia* OR hyperglycemia* OR 'glucose intoleran*' OR 'insulin resistan*' OR 'metabolic syndrome'):ab,ti,kw
#25 MeSH descriptor: [Comorbidity] explode all trees
#26 ((('long term' OR 'long-term' OR 'late') NEXT/2 effect*) OR comorbid* OR 'co-morbid*' OR polymorbid* OR 'poly-morbid*' OR multimorbid* OR 'multi-morbid*' OR (('concomitant' OR coexist* OR 'co-exist*' OR 'chronic' OR 'long-term' OR 'long term') NEXT/2 (illness* OR condition* OR disease*))):ab,ti,kw
#27 #17 OR #18 OR #19 OR #20 OR #21 OR #22 OR #23 OR #24 OR #25 OR #26
#28 MeSH descriptor: [Control Groups] explode all trees
#29 ('general' OR 'community' OR 'cancer-free' OR 'cancer free' OR 'non-cancer' OR (('free' OR 'no' OR 'non' OR 'not' OR 'without') NEAR/5 (cancer* OR carcinoma* OR tumor* OR tumour* OR neoplasm* OR malign* OR oncolog* OR adenocarcinoma* OR choriocarcinoma* OR leukaemia* OR leukemia* OR lymphoma* OR sarcoma* OR teratoma* OR myeloma* OR 'melanoma' OR melanoma*)) OR 'health*' OR 'compar*' OR 'control*' OR 'sibling*'):ab,ti,kw
#30 #28 OR #29

#31 MeSH descriptor: [Incidence] explode all trees
#32 MeSH descriptor: [Risk] explode all trees
#33 MeSH descriptor: [Odds Ratio] explode all trees
#34 inciden* OR risk* OR ratio*:ab,ti,kw
#35 #31 OR #32 OR #33 OR #34
#36 MeSH descriptor: [Cohort Studies] explode all trees
#37 MeSH descriptor: [Longitudinal Studies] explode all trees
#38 MeSH descriptor: [Follow-Up Studies] explode all trees
#39 MeSH descriptor: [Prospective Studies] explode all trees
#40 MeSH descriptor: [Retrospective Studies] explode all trees
#41 ('cohort' OR 'longitudinal' OR 'follow-up' OR 'follow up' OR 'prospective' OR 'retrospective' OR 'observational') NEAR/5 (‘study’ OR ‘studies’ OR design* OR research* OR trial*)
#42 #36 OR #37 OR #38 OR #39 OR #40 OR #41
#43 #16 AND #27 AND #30 AND #35 AND #42

Web of Science

#1 TS=(((cancer* OR carcinoma* OR tumor* OR tumour* OR neoplasm* OR malign* OR oncolog* OR adenocarcinoma* OR choriocarcinoma* OR leukaemia* OR leukemia* OR lymphoma* OR sarcoma* OR teratoma* OR myeloma* OR 'melanoma' OR melanoma*) AND surviv*) OR ((cancer* OR carcinoma* OR tumor* OR tumour* OR neoplasm* OR malign* OR oncolog* OR adenocarcinoma* OR choriocarcinoma* OR leukaemia* OR leukemia* OR lymphoma* OR sarcoma* OR teratoma* OR myeloma* OR 'melanoma' OR melanoma*) NEAR/5 (patient* OR 'man' OR 'men' OR 'woman' OR 'women' OR child*)))
#2 TS=(diabet* OR DM OR IDDM OR NIDDM OR "diabetes mellitus" OR "diabetes mellitus, type 1" OR "type 1 diabetes mellitus" OR "type 1 diabetes" OR T1DM OR "diabetes mellitus, type 2" OR "type 2 diabetes mellitus" OR "type 2 diabetes" OR T2DM OR "glucose intoleran*" OR "insulin resistan*" OR hyperglycaemia* OR hyperglycemia* OR "metabolic syndrome")
#3 TS=(((“long term” OR “long-term” OR “late”) NEAR/2 effect*) OR comorbid* OR “co-morbid*” OR polymorbid* OR “poly-morbid*” OR multimorbid* OR “multi-morbid*” OR ((“concomitant” OR coexist* OR “coexist*” OR “chronic” OR “long-term” OR “long term”) NEAR/2 (illness* OR condition* OR disease*)))
#4 #2 OR #3
#5 TS=(“general” OR “community” OR “cancer-free” OR “cancer free” OR “non-cancer” OR ((“free” OR “no” OR “non” OR “not” OR “without”) NEAR/5 (cancer* OR carcinoma* OR tumor* OR tumour* OR neoplasm* OR malign* OR oncolog* OR adenocarcinoma* OR choriocarcinoma* OR leukaemia* OR leukemia* OR lymphoma* OR sarcoma* OR teratoma* OR myeloma* OR 'melanoma' OR melanoma*)) OR health* OR compar* OR control* OR sibling*)
#6 TS=(inciden* OR risk* OR ratio*)
#7 TS=((“cohort” OR “longitudinal” OR “follow-up” OR “follow up” OR “prospective” OR “retrospective” OR “observational”) NEAR/5 (“study” OR “studies” OR design* OR research* OR trial*))
#8 #1 AND #4 AND #5 AND #6 AND #7

Table S1. Summary of additional characteristics of studies included in meta-analysis.

Study (First Author, Year of Publication, And Country)	Age of Cancer Patients (Years)	Index Date of Cancer Patients	Method of Diagnosis		
			Cancer	Diabetes	
				Type	Incident Diabetes
Khan, 2011 (UK) [35]	Mean ± SD at survey: Breast: 66.9 ± 12.3 Colorectal: 74.1 ± 10.9 Prostate: 76.1 ± 8.1	1st September 2003; Mean years from diagnosis to index date (± SD) is 10.1 ± 7.9	Records from the UK GPRD	Not differentiated	Read or OXMIS codes from the UK GPRD; newly diagnosed within the analysis period
Landis, 2011 (UK) [36]	Mean ± SD at index date: 62 ± 12	The date of the first hospitalization; Incidence rates and hazard ratios were stratified by time since diagnosis.	ICD-9-CM codes from PHARMO RLS which is linked to ECR of the Comprehensive Cancer Centre South	Not differentiated	ICD-9-CM codes and/or ATC codes from PHARMO RLS which is linked to ECR of the Comprehensive Cancer Centre South; after the cancer diagnosis
Danese, 2012 (USA) [37]	≥66 at cancer diagnosis	The first day of the SEER month and year of cancer diagnosis	SEER data	Not differentiated	ICD-9 from Medicare claims data; after the index date
Li, 2012 (USA) [38]	Mean ± SD at cancer diagnosis: 72 ± 9; ≥18 at cancer diagnosis	Date of the Prostate cancer diagnosis	Clinical records from GPRD	T2DM	Read and OXMIS Codes from GPRD; after index date
Lipscombe, 2012 (Canada) [39]	Mean ± SD at cancer diagnosis: 68.49 ± 9.17; ≥55 at cancer diagnosis	Cohort entry date was defined as the date of breast cancer diagnosis	Data from OCR, the Canadian Institute for Health Information Discharge Abstract database, the Registered Persons Database, ODD and the Ontario Health Insurance Plan database	Not differentiated	Data from OCR, the Canadian Institute for Health Information Discharge Abstract database, the Registered Persons Database, ODD and the Ontario Health Insurance Plan database; start from cohort entry date

**Study (First Author, Year of Publication, And Country)**	**Age of Cancer Patients (Years)**	**Index Date of Cancer Patients**	**Method of Diagnosis**		
			**Cancer**	**Diabetes**	
				**Type**	**Incident Diabetes**
Stålberg, 2012 (Sweden) [40]	Median at cancer diagnosis: 63	Diagnosis date for ovarian cancer plus 30 days	ICD-7 from CaCom	Not differentiated	ICD-9 and ICD-10 from CaCom; starting at 30 days after the ovarian cancer diagnosis
van Herk-Sukel, 2012 (The Netherlands) [41]	Mean ± SD at cancer diagnosis: 56 ± 20	Date of first soft tissue sarcomas pathology specimen (cohort entry date)	ICD-9-CM, morphology ICD-O-3 codes and ATC codes from the PALGA-PHARMO RLS linkage	Not differentiated	ICD-9-CM and ATC codes from the Dutch National Medical Register and the community (outpatient) pharmacy database; after cohort entry date
Chia, 2013 (USA) [42]	≥66 at cancer diagnosis	The first day of the month of the ovarian cancer diagnosis	From SEER-Medicare database	Not differentiated	ICD-9 from SEER-Medicare database; after index date
Jordan, 2014 (USA) [43]	≥65 at cancer diagnosis	Date of breast cancer diagnosis	ICD-Oncology codes, ICD-9 or -10 codes and CPT codes from medical record of six of CRN members: GHC, KPSC, L, HF, HP, F	Not differentiated	Medical record of six of CRN members: GHC, KPSC, L, HF, HP, F; during years 6–15 after index date
Sun, 2014 (Taiwan, China) [44]	Mean ± SD at index date: 50.43 ± 11.27	The first prescription of tamoxifen	ICD-9-CM from the registry for CIPD	T2DM	ICD-9-CM from the registry for CIPD; after the index date
Li, 2015 (China) [45]	20–80 at cancer diagnosis	The date of breast cancer diagnosis	Data from hospital pathological examination	T2DM	The medical history and prescription records which were obtained by doctors during interview; after diagnosis
Ording, 2015 (Denmark) [46]	Median at index date: 66.4	5 years after diagnosis	Data from DCR	Not differentiated	ICD-8 or ICD-10t from DNRP; after the index date
Chang, 2016 (China) [47]	Mean ± SD at cancer diagnosis: 29.9 ± 16.4	The date of AML diagnosis	ICD-9-CM from NHIRD	Not differentiated	ICD-9-CM from NHIRD; after index date
Chao, 2016 (USA) [48]	0-18 at cancer diagnosis	5 years after cancer diagnosis	Data from KPSC's SEER-affiliated cancer registry	Not differentiated	ICD-9 codes from KPSC's SEER-affiliated cancer registry; after the index date

Study (First Author, Year of Publication, And Country)	Age of Cancer Patients (Years)	Index Date of Cancer Patients	Method of Diagnosis		
			Cancer	Diabetes	
				Type	Incident Diabetes
Crawley, 2016 (UK) [49]	Mean ± SD at cancer diagnosis: 74.4 ± 8.4	Follow up started on 1st January 2006; Time of diabetes was calculated from ADT exposure	Data from NPCR of Sweden; should receive primary or secondary ADT (from NPCR or the National Prescribed Drug Register using ATC codes)	T2DM	Data from the National Prescribed Drug Register using ATC codes; first prescription after 1st Jan. 2006
Hashibe, 2016 (USA) [50]	Median at cancer diagnosis: 31; ≥15 at cancer diagnosis	Date of breast cancer diagnosis	ICD-O-3 code from UPDB	Not differentiated	ICD-9 and CPT codes from UPDB; diagnosed 5 years after the testicular cancer diagnosis
Lowe, 2016 (USA) [51]	≥66 at cancer diagnosis	The first day of the month of gastric cancer diagnosis	From SEER-Medicare database	Not differentiated	ICD-9 from SEER-Medicare database; after index date
Santorell, 2016 (USA) [27]	>65 at index date	The earliest hormonal therapy fill date	From SEER-Medicare linked data; should fill a prescription for an AI or for tamoxifen	Not differentiated	ICD-9 from SEER-Medicare linked data; after treatment start date
Singh, 2016 (Canada) [52]	Mean ± SD at cancer diagnosis: 68.00 ± 13.19; 20-105 at cancer diagnosis	The date of diagnosis of CRC	Data from OCR, the Canadian Institute for Health Information Discharge Abstract database, the National Ambulatory Care Reporting System databases, the Registered Persons Database, ODD and the Ontario Health Insurance Plan database	Not differentiated	Data from OCR, the Canadian Institute for Health Information Discharge Abstract database, the National Ambulatory Care Reporting System databases, the Registered Persons Database, ODD and the Ontario Health Insurance; start from index date
Blackburn, 2017 (USA) [53]	Median at cancer diagnosis: 46	Thyroid cancer diagnosis date	ICD-9 from UPDB	Not differentiated	ICD-9 from UPDB; from cancer diagnosis

Study (First Author, Year of Publication, And Country)	Age of Cancer Patients (Years)	Index Date of Cancer Patients	Method of Diagnosis		
			Cancer	Diabetes	
				Type	Incident Diabetes
Hwangbo, 2018 (South Korea) [25]	20-70 on the date of their first health screening examination	Non-cancer and non-diabetes population was included in the study on the date of their first health screening examination; Hazard ratio for diabetes was stratified by time since diagnosis.	ICD-10, and the Korean Drug and Anatomical Therapeutic Chemical Codes, from NHIS-NSC	T2DM	ICD-10, from NHIS-NSC; after the first 31 days since cancer diagnosis
Lega, 2018 (Canada) [54]	Mean ± SD at cancer diagnosis: 10.7 ± 6.8; <21 at cancer diagnosis	the date of 1-year survival following cancer diagnosis	ICCC-3 from OCR	Not differentiated	ICD-9 from OHIP database and ODD; after index date
Ng (BC), 2018 (Australia) [55]	Age at index date: mostly ≥55	When therapy started	World Health Organization Anatomical Therapeutic Chemical code and PBS schedule item codes from PBS	Not differentiated	World Health Organization Anatomical Therapeutic Chemical code and PBS schedule item codes from PBS; after initiation of endocrine therapy
Ng (PC), 2018 (Australia) [56]	Age at index date: mostly ≥65	The first dispensing date of ADT	World Health Organization Anatomical Therapeutic Chemical code and PBS schedule item codes from PBS	Not differentiated	World Health Organization Anatomical Therapeutic Chemical code and PBS schedule item codes from PBS; after initiation of ADT
Dhopeshwarkar, 2019 (USA) [57]	Mean ± SD at cancer diagnosis: 77.3 ± 6.96	The earliest AML diagnosis date	ICD-O-3/WHO recode (SEER) and ICD-9 (Medicare claims data)	T2DM	ICD-9 codes from Medicare claims data; after index date
Hawkins, 2019 (USA) [58]	Mean ± SD at cancer diagnosis: 63.7 ± 14.0	Follow-up time: from cancer diagnosis date	ICD-9 from UPDB	Not differentiated	ICD-9 from UPDB; after cancer diagnosis

Study (First Author, Year of Publication, And Country)	Age of Cancer Patients (Years)	Index Date of Cancer Patients	Method of Diagnosis		
			Cancer	Diabetes	
				Type	Incident Diabetes
Ng, 2019 (Australia) [59]	≥18 at cancer diagnosis	The date of breast cancer diagnosis	ICD-O-3, ICD-9, ICD-10 and Drug identification number assigned by Health Canada linked to ATC codes from BC Cancer Registry/Breast Cancer Outcome Unit database, BCMOH MSP Registry, BCMOH MSP claims database, CIHI Discharge Abstract Database, BCMOH PharmaNet database and BC Vital Statistics Agency	Not differentiated	ICD-9, ICD-10 and Drug identification number assigned by Health Canada linked to ATC codes from BC Cancer Registry/Breast Cancer Outcome Unit database, BCMOH MSP Registry, BCMOH MSP claims database, CIHI Discharge Abstract Database, BCMOH PharmaNet database and BC Vital Statistics Agency; after the index date
Accordino, 2020 (USA) [60]	≥66 at cancer diagnosis	The date of diagnosis	From SEER-Medicare database	Not differentiated	ICD9 code and/or a prescription claim after 2007 for a DM medication using HCPCS codes from SEER-Medicare database; after the index date
Bigelow, 2020 (USA) [61]	Median at diagnosis: 72; ≥66 at cancer diagnosis	1 year before diagnosis; Time to diabetes was calculated from diagnosis to the date of the first claim for diabetes	ICD-O-3 from SEER data	Not differentiated	ICD-9 from Medicare claims data; after 30 days from cancer diagnosis
Chao, 2020 (USA) [62]	Mean ± SD at cancer diagnosis: 31.3 ± 6.5; 15-39 at cancer diagnosis	2 years after cancer diagnosis	Data from KPSC	Not differentiated	ICD-9 code, laboratory data or medication data from KPSC; diagnosed after the index date
Kim, 2020 (USA) [63]	Mean ± SD at cancer diagnosis: 60.5 ± 12.8; ≥18 at cancer diagnosis	Cancer diagnosis date	ICD-O-3 from SEER Utah Cancer Registry and UPDB	Not differentiated	ICD-9 from SEER Utah Cancer Registry and UPDB; after endometrial cancer diagnoses

Study (First Author, Year of Publication, And Country)	Age of Cancer Patients (Years)	Index Date of Cancer Patients	Method of Diagnosis		
			Cancer	Diabetes	
				Type	Incident Diabetes
Markus, 2020 (Israel) [64]	Mean ± SD at cancer diagnosis: MM: 64.5 ± 12.5 SMM: 67.8 ± 9.4	Baseline: the time of diagnosis	Medical records of patients treated at TASMC according to the criteria of the International Myeloma Working Group	Not differentiated	Medical records of patients treated at TASMC; after baseline
Wang, 2020 (Taiwan, China) [65]	Mean (SD) at cancer diagnosis: 58.6 (9.1); ≥55 at cancer diagnosis	The date breast cancer was first diagnosed	ICD-9-CM from NHIRD	Not differentiated	ICD-9 from NHIRD; after cancer diagnosis
Lin, 2021 (Taiwan, China) [26]	Mean ± SD at cancer diagnosis: 73.6 ± 9.7; ≥18 at cancer diagnosis	The first diagnosis date	ICD-9-CM from LHID 2000	type II or unspecified type	ICD-9-CM from LHID 2000; after index date

Abbreviation: ADT, Androgen Deprivation Therapy; AML, Acute myeloid leukemia; CRC, colorectal cancer; MM, multiple myeloma; SCCHN, squamous cell carcinoma of the head and neck; SD, Standard Deviation; SMM, smoldering multiple myeloma; DM, diabetes mellitus; T2DM, type 2 diabetes mellitus;

Abbreviation of coding: ATC, the Anatomical Therapeutic Chemical; ICD-9, International Classification of Diseases, Ninth Revision; ICD-9-CM, International Classification of Diseases, Ninth Revision, Clinical Modification; ICD-10, International Classification of Diseases, Tenth Revision; ICD-O-3, the International Classification of Diseases for Oncology, third edition; ICD-O-3/WHO, International Classification of Diseases for Oncology, 3rd edition/World Health Organization; ICCC-3, International Classification of Childhood Cancer, Third Edition; CPT, Current Procedural Terminology; HCPCS, Healthcare Common Procedure Coding System;

Abbreviation of registries: PHARMO RLS, the PHARMO medical record linkage system; ECR, the Eindoven cancer registry; GPRD, General Practice Research Database; SEER, the Surveillance, Epidemiology, and End Results program; OCR, the Ontario Cancer Registry; ODD, the Ontario Diabetes Database; CaCom, the Cancer and Co-morbidity Database; PALGA, the Dutch Nationwide Network and Registry of Histo- and Cytopathology; CRN, the Cancer Research Network; GHC, Group Health Cooperative; KPSC, Kaiser Permanente Southern California; L, Lovelace; HF, Henry Ford Health System; HP, HealthPartners; F, Fallon Community Health Plan; CIPD, the Catastrophic Illnesses Patient Database; DCR, the Danish Cancer Registry; DNRP, the Danish National Registry of Patients; NHIRD, the Taiwanese National Health Insurance Research Database; NPCR, the National Prostate Cancer Register; UPDB, the Utah Population Database; NHIS-NSC, the National Health Insurance Service-National Sample Cohort; OHIP, the Ontario Health Insurance Plan; PBS, the Pharmaceutical Benefits Scheme; BCMOH MSP, BC Ministry of Health Medical Services Plan; CIHI, Canadian Institute for Health Information; TASMC, Tel Aviv Sourasky Medical Center; LHID, the Longitudinal Health Insurance Database.
